# Collapse from Perforation of Cæcum or Appendix

**Published:** 1892-02

**Authors:** 


### Editorial.

### COLLAPSE FROM PERFORATION OF CÆCUM OR APPENDIX.

The consideration of disorders in the right iliac region having occupied a prominent place in medical literature for some years, it is a notable fact that little attention has been directed to one class of cases, requiring early recognition and prompt action for their relief.

In localized inflammation and perforation of the cæcum or appendix, collapse ensues without premonitory constitutional disturbance, and the prostration of the vital forces is so sudden that death occurs in a few hours, or, at most, a few days after the first indications of trouble.

Much has been said in regard to the relative advantages of medical treatment and surgical measures in typhlitis and appendicitis, which implies a comparatively slow progress of inflammation in the tissues of the cæcum or appendix vermiformis under certain conditions of the structures involved. But in the fulminating developments connected with perforation and discharge of fæcal matter into the peritoneal cavity, there can be no question in regard to the urgency of surgical interference, and the great disideratum is to make timely diagnosis with a view to prompt action.

The insidious character of the symptoms, like those apparently mild cases of typhoid, or, more properly, enteric fever, in which a small perforation of the ileum leads to fatal collapse is calculated to mislead the practitioner, whether physician or surgeon, as to the nature of the change which takes place

Stangely enough, the local manifestations are of so slight a character as scarcely to have attracted the attention of the patient, and perhaps the regular family attendant has not been called until perforation has actually led to marked vital depression. If not on the look out for the true cause, the premonitory local disturbance may be ignored in seeking to arouse the patient from the incipient collapse; and it is only after failure to relieve the prostration that a correct history of the incipient stage of the disorder leads to a proper diagnosis. A surgeon is then summoned, with the intimation such as the writer has received on several occasions, to come prepared for a laparotomy, when the opportunity has already passed for affording any relief by surgical interference in the case.

The important matter of determining in advance of perforation what is the nature of the case, cannot be decided when the patient has not been seen by physician or surgeon; but in a large proportion of cases the patient has come under professional care while the local modifications admit of examination. It is, then, at this stage of the inflammatory action that we need light upon the seat of disorder and the structural changes which are in progress. Those who have had most experience in diagnosing the disturbances of the right iliac region are not prepared to lay down any invariable rules by which these points can be settled. It is simply an impossibility to define what is the exact pathological state of the parts involved, and hence the only available mode for a satisfactory diagnosis is an exploratory operation.

The writer holds the same view now that was presented by him in a paper presented to the surgical section of the American Medical Association in 1886, on "Surgical Relations of the Ileo-Cæcal Region." It was then set forth that "in cases of doubtful diagnosis, presenting reasonable grounds for the conviction that there exists a disease calling for operative interference, exploratory laparotomy should be resorted to at an early stage, after other modes of treatment have failed to afford relief; and the consequences of this opening the abdominal cavity, where operations upon the contained organs are not undertaken, are not generally serious." Of course, no time should be lost when the evidence of perforation exists; but the point here made is that this result should be anticipated whenever the indications of inflammation of cæcum or the appendix afford a sufficient reason to expect that perforation of their coats may ensue. The consequences of such a lesion are of such a grave nature, not simply by the peritonitis and necrosis of adjacent tissues, but from the sudden supervention of collapse, that considerable responsibility attaches to delay in ascertaining the exact conditions involved by a surgical procedure. Those precautionary measures, which may avert a great calamity, become conservatism instead of being classed as rashness, and temerity, with discretion, is the highest and noblest attribute of the surgeon.

J. McF. G.

